# Incidence and Remission of Atopic Dermatitis in a German Birth Cohort

**DOI:** 10.1001/jamanetworkopen.2025.44324

**Published:** 2025-12-08

**Authors:** Chu-Wei Hung, Stephanie Roll, Katja Icke, Linus Grabenhenrich, Julia Fricke, Volker Wahn, Antje Schuster, Oliver Nitsche, Armin Grübl, Christoph Müller, Margitta Worm, Ulrich Wahn, Susanne Lau, Thomas Keil

**Affiliations:** 1 Institute of Social Medicine, Epidemiology and Health Economics, Charité–Universitätsmedizin Berlin, corporate member of Freie Universität Berlin and Humboldt Universität zu Berlin, Berlin, Germany; 2 Institute for Biostatistics and Informatics in Medicine and Ageing Research, Rostock University Medical Center, Rostock, Germany; 3 Department for Methods Development, Research Infrastructure and Information Technology, Robert Koch Institute, Berlin, Germany; 4 Staff Unit for Municipal Health Strategies for the City of Freiburg and the District of Breisgau-Hochschwarzwald, Freiburg, Germany; 5 Pediatric Respiratory Medicine, Immunology and Critical Care Medicine, Charité–Universitätsmedizin Berlin, corporate member of Freie Universität Berlin and Humboldt Universität zu Berlin, Berlin, Germany; 6 Department of Pediatrics, University Hospital Düsseldorf, Düsseldorf, Germany; 7 Section of Pediatric Pulmonology, Allergology, and Cystic Fibrosis, Center for Pediatrics and Adolescent Medicine, Universitätsmedizin Mainz, Mainz, Germany; 8 Department of Pediatrics, Technical University Munich, Munich, Germany; 9 Center for Pediatrics and Adolescent Medicine, University of Freiburg, Faculty of Medicine, Freiburg, Germany; 10 Division of Allergy and Immunology, Department of Dermatology, Venereology and Allergology, Charité–Universitätsmedizin Berlin, corporate member of Freie Universität Berlin and Humboldt Universität zu Berlin, Berlin, Germany; 11 German Center for Child and Adolescent Health, Partner Site Berlin, Berlin, Germany; 12 Institute of Clinical Epidemiology and Biometry, University of Würzburg, Würzburg, Germany; 13 State Institute of Health I, Bavarian Health and Food Safety Authority, Erlangen, Germany

## Abstract

**Importance:**

Patterns of incidence and factors associated with remission of atopic dermatitis (AD) from birth to adulthood remain understudied.

**Objectives:**

To examine incidence of AD, its remission probability and trajectories, and early-life factors associated with it in a prospective study from birth to 30 years.

**Design, Setting, and Participants:**

This cohort study used data from the population-based Multicenter Allergy Study (MAS), which recruited full-term newborns from 5 German cities in 1990. Generally healthy newborns were enrolled; the sample was slightly enriched for allergy risk, with more than a third of participants born with 2 parents or siblings with allergies. Until age 30 years, participants were assessed by parental or self-report questionnaires (20 times) and clinical evaluations, including serum samples (9 times).

**Exposures:**

Age, sex, age of AD onset, parental allergy status, early-life sensitization against common food and aero-allergens, rhinitis during ages 3 to 5 years, and asthma at age 6 years.

**Main Outcomes and Measures:**

AD was determined by standardized questionnaire and doctor’s diagnoses. AD remission was defined as being symptom-free for at least 12 months.

**Results:**

Among 1314 recruited participants, 707 (57%) had 1 or 2 allergic parents. Overall, 482 (37%) participated at the 30-year follow-up. The cumulative incidence of AD during the first 5 years was 57% (402 participants) with 1 or 2 allergic parents and 43% (225 participants) with nonallergic parents. The yearly rate of new AD cases declined from age 2 until 13 years and increased slightly thereafter. Among 529 participants with AD onset in the first 5 years of life, 459 (87%) reported no symptoms at age 20 and 30 years. Using data from 658 participants, 4 remission trajectories were identified: early childhood, early school age, substantial adolescence, and partial adulthood remission. Onset within first 2 years of life (adjusted risk ratio [aRR], 1.59; 95%- CI, 1.12-2.26), female sex (aRR, 1.56; 95% CI, 1.23-2.00), early allergic sensitization against at least 1 common aero- or food allergen (aRR, 1.95; 95% CI, 1.25-3.05), asthma at age 6 years (aRR, 2.22; 95% CI, 1.70-2.89), and allergic rhinitis at ages 3 to 5 years (aRR, 1.54; 95% CI, 1.21-1.95) were associated with the persistent phenotype.

**Conclusions and Relevance:**

In this cohort study of children with allergic diseases, AD before school age was generally associated with a favorable long-term prognosis. However, remission was less likely in female participants, those with onset before age 2 years, those with specific serum antibodies to common allergens before school age, or those with allergic comorbidity, highlighting the need for increased clinical attention and further investigation into underlying mechanisms.

## Key Points

**Question:**

Will children with atopic dermatitis outgrow the disease, and what early-life factors are associated with its remission during the transition to adulthood?

**Findings:**

In this cohort study with 1314 participants, 87% of participants with atopic dermatitis onset before school age experienced remission by age 30 years. Male children, those with onset after the age of 2 years, and those without specific antibodies to common aero- or food allergens or allergic respiratory symptoms early in life were more likely to become free of symptoms by adulthood.

**Meaning:**

The findings of this study suggest that most children with atopic dermatitis experience symptom resolution by adulthood, with later onset, male sex, and no allergic reactions to common allergens before age 5 years or comorbid allergic conditions being associated with a higher chance of remission.

## Teaser

This cohort study evaluates the trajectory of and early-life factors associated with atopic dermatitis from birth to age 30 years.

## Introduction

Atopic dermatitis (AD) is a chronic disease often starting very early in life characterized by skin barrier dysfunction and type 2 inflammation.^1^ The clinical course can vary, with potentially fluctuating symptoms over the lifespan. Until now, understanding the natural history of AD from birth to adulthood is still incomplete, especially given that long-term prospective investigations are rare.^2,3,4^ While clinicians often observe that AD resolves by early school age, recent longitudinal studies suggested that some affected children may experience persistent AD up to adulthood.^5,6,7^ However, only a few population-based studies have investigated the occurrence and determinants of AD remission prospectively from birth to adulthood.^6,8,9,10^

As the oldest German and second oldest European birth cohort study on allergies, the Multicenter Allergy Study (MAS) has contributed to expanding knowledge on allergies up to age 20 years.^11,12,13^ However, an update on disease status in adulthood and in particular the investigation of factors associated with AD remission is lacking. MAS will help clinicians and affected families address relevant questions about disease progression, early-life determinants, and predicting adult diseases from childhood conditions. Furthermore, it aids in identifying risk groups with prolonged disease and poorer prognosis as candidates for early anti-inflammatory intervention by systemic therapy, potentially modifying the progression not only of AD but also of associated allergic comorbidity.^14^

This study aimed to determine the prevalence and incidence of AD, along with its remission probability and trajectories among MAS birth cohort participants from birth to age 30 years. We also aimed to investigate possible factors associated with AD remission, including age of onset, sex, parental allergy status, early-life sensitization to common allergens, and respiratory allergies early in life.

## Methods

### Study Design and Population

The prospective German birth cohort study MAS launched in 1990 and collected data at 20 follow-up assessments over 30 years. Ethical approval was obtained from local ethics committees of participating hospitals. Written parental consent was obtained after birth. This report adheres to the Strengthening the Reporting of Observational Studies in Epidemiology (STROBE) reporting guideline for cohort studies.

Parents of 7609 newborns from 6 obstetrics departments in 5 German cities were screened. Families declining questionnaires or lacking cord blood samples were excluded. Eligible participants were selected using a risk-enriched sampling strategy. Infants with a high risk of allergy (defined in 1989 having 2 allergic parents or siblings and/or cord blood immunoglobulin E [IgE] levels ≥0.9 kU/L) were oversampled; others were randomly selected from the base population. Among screened newborns, only 973 (13%) were at high risk of allergy. Recruitment yielded 1314 full-term newborns who did not require ventilation or intensive care, including 499 (38%) classified as high-risk, while 815 (62%) had a low or moderate allergy risk.^15,16^

### Data Collection

Parents were interviewed during 6 clinical evaluations in the first 2 years of the child’s life, then assessments took place yearly from ages 3 until 13 years and at 15, 20, and 30 years. Serum samples were obtained at ages 1, 2, 3, 5, 6, 7, 10, 13 and 20 years. Information regarding parental history of allergy, allergic symptoms, and diagnoses was collected using a standardized questionnaire, including validated allergy questions from the International Study of Asthma and Allergies in Childhood project.^17^

### Outcome Variable

We defined AD cases based on parent-reported symptoms, a parent-reported previous physician’s diagnosis of AD, and the assessment by the study physician during the visits, with differing case definition criteria at different follow-ups due to varying assessment methods throughout the study (eTable 1 in Supplement 1). AD cases occurring at ages 3 and 6 months were counted as cases at age 1 year, and cases reported at the 18-month follow-up were counted into age 2 years. Current AD was defined as having AD symptoms within the past 12 months, including both incident and prevalent cases. We defined AD remission when participants who ever had AD reported no more AD symptoms (ie, itchy rash in specific typically affected areas) for the last 12 months. To describe AD status in adulthood, we defined persistent or relapsing AD when participants reported AD symptoms at ages 20 or 30 years, and we defined remission at ages 20 and 30 years when AD symptoms were absent at both follow-up assessments or when participants had missing data at one assessment, while no AD symptoms were indicated at the other follow-up assessment.

### Exposure Variables

For the variable age of AD onset, we defined early-onset AD when symptoms occurred for the first time between birth and age 5 years, while we defined late-onset AD when the first symptoms occurred after the age of 5 years. To investigate the association between the age of AD onset and the risk of AD or factors associated with remission, we further categorized AD onset into 2 to 4 groups, based on various analyses (eMethods 1 in Supplement 1). Age was expressed both as a continuous variable and categorized by life stage (childhood, 1-5 years; early school age, 6-10 years; prepuberty and puberty, 11-15 years; and late adolescence and early adulthood, 20-30 years) depending on the specific research question. Parental allergy status was categorized as yes if at least 1 parent had self-reported allergic rhinitis, asthma, or AD and no if both were nonallergic. Early sensitization against common allergens was determined based on serum samples collected at ages 1, 2, 3, and 5 years. A composite dichotomous variable, early allergic sensitization, was created using information across all 4 time points. Additional factors, such as rhinitis during age 3 to 5 years and asthma at age 6 years, were explored in relation to AD remission trajectories (eMethods 2 in Supplement 1).

### Statistical Analysis

One-year prevalence of AD was computed by dividing the number of AD cases by the number of participants at the respective follow-up period. Cumulative incidence was determined as the proportion of participants who developed AD up to a given point in time. One-year incidence was computed by dividing the new cases since the last 12 months by the population at risk (without those who already had AD) during the respective ages of interest. Since MAS is a risk-enriched cohort and parental allergy is a strong risk factor, we further stratified incidence by parental allergy status.

To evaluate the association between current AD and age of onset over time, we used generalized linear mixed model (GLMM) with logit link, accounting for the longitudinal variation of individuals by assigning participant identification as random intercept. A priori confounders included life stage, sex, and parental allergy status.

We analyzed AD remission over time using 2 approaches for participants with early-onset AD: (1) treating all participants as a homogeneous group to calculate the estimated probability of AD remission based on GLMM and (2) assuming heterogeneous remission trajectories (ie, phenotypes) to assess their association with potential determinants using a growth mixture model (GMM)—a statistical method that identifies hidden subgroups in longitudinal data, each with its own average trajectory and individual variation^18,19,20^ (eMethods 3 in Supplement 1). We finally explored the association between early-life factors (exploratory variables) and remission trajectories (outcomes) using log-binomial regression to calculate risk ratios.

Due to the exploratory nature of the study and the potential for nonrandom missingness, we did not impute the outcome or exposure variables. Pairwise deletion was automatically executed in regression analyses. All observations remained in the analysis, even with missing data at some time points. Sensitivity analyses were conducted (eTable 4 and eTable 7 in Supplement 1). All *P* values are 2-sided and are interpreted in an explorative way only. We performed analyses using R version 4.2.0 (R Project for Statistical Computing) with the lme4 package for GLMM and the lcmm package for GMM.^21,22^

## Results

### Characteristics of Study Population

Of the 1314 participants recruited for MAS (630 [48%] female; 707 [57%] with ≥1 allergic parents; mean [SD] follow-up appointments, 13 [6]; less than 5% with non-German backgrounds), 658 individuals (50.1%) experienced early-onset AD (ie, until the age of 5 years). Thirty-one participants (2.4%; 20 women) reported AD onset at age 20 or 30 years. Most of those with early-onset AD were sensitized early (ie, first 5 years) against at least 1 common allergen, had a parental history of allergies, and were male (eTable 2 in Supplement 1). On average, individuals without AD participated in fewer follow-up assessments compared to those with AD. At ages 20 and 30 years, data were available for 942 (72%) and 482 (37%) participants, respectively (eFigure 1 in Supplement 1); factors related to attrition in MAS have been reported previously.^11,23^

### Prevalence and Incidence

The prevalence of AD consistently exceeded 20% from age 1 year to age 5 years. Subsequently, it decreased from approximately 15% to 10% between the ages of 6 and 20 years. At age 30 years, the prevalence rose again (eFigure 1 in Supplement 1).

**Figure 1.**
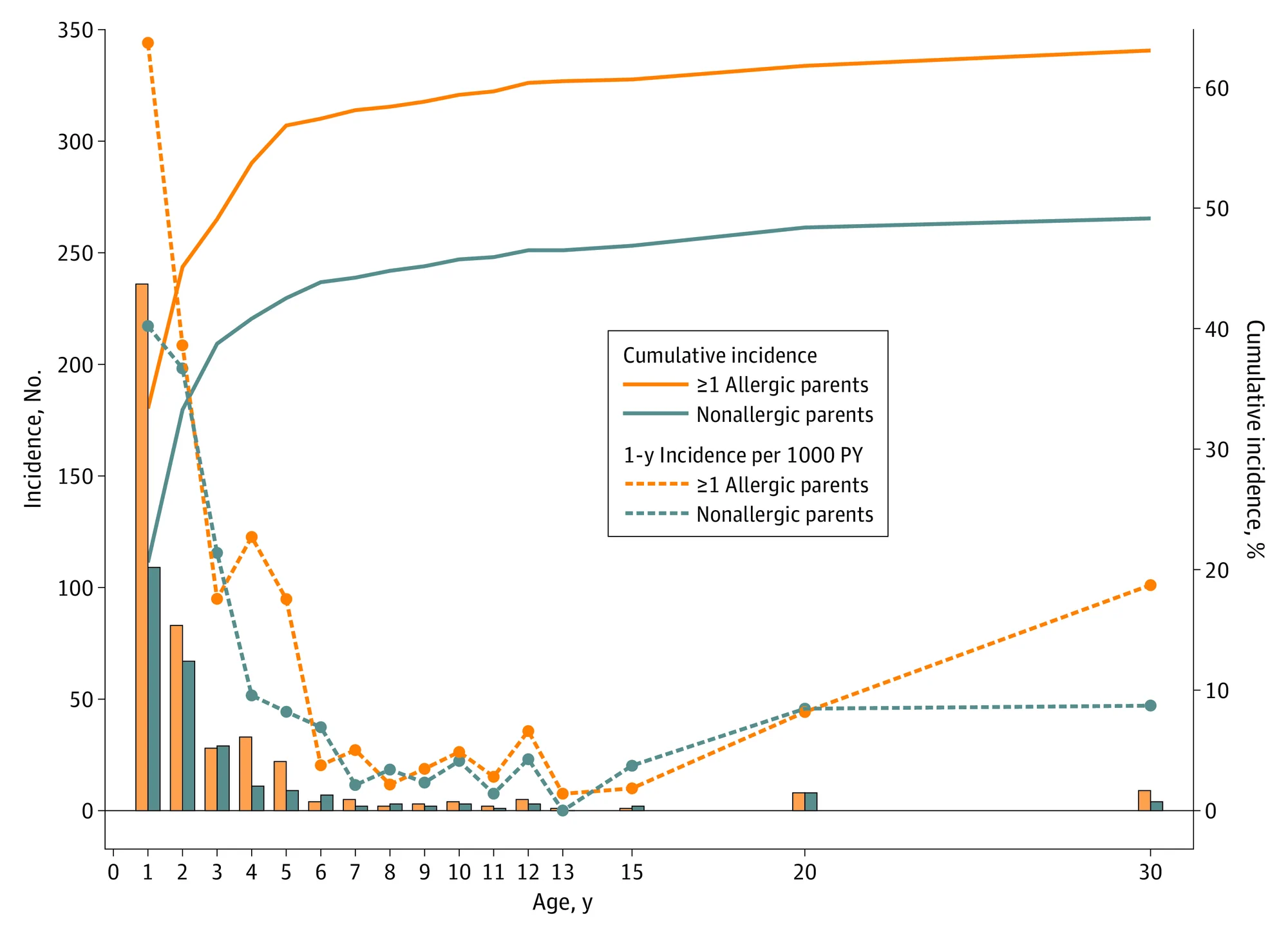
Incidence of Atopic Dermatitis (AD) by Parental Allergy Status in the German MAS Birth Cohort Study The orange bars indicate new cases of AD in children with at least 1 allergic parent, and the blue bars indicate new cases of AD in children with nonallergic parents (see left y-axis for numbers). Cumulative incidence (solid lines; right y-axis) had 707 and 529 participants recruited at birth as the denominators for children with at least 1 allergic parent and those with nonallergic parents, respectively. The 1-year incidence rate (dashed lines; left y-axis) was calculated by new cases from the year of the follow-up age (past 12 months) divided by the population at risk at the respective follow-up age; the incidence rate can be interpreted as the number of new AD cases per 1000 person-years (PY).

Among both risk groups (with and without allergic parents), the cumulative incidence exhibited the steepest increase between ages 1 and 2 years, followed by a slower rise thereafter (Figure 1). For example, among children with nonallergic parents (dashed blue line), the 1-year incidence at 1 year was 217.1; at 2 years, 198.2; at 10 years: 22.2; at 15 years, 20.2; and at 30 years, 47.1. One-year incidence rates displayed similar trends, peaking before age 3 years, and reaching their lowest point at age 13 years. Beyond this age, a gradual increase in AD incidence was observed. Overall, the AD incidence seemed to be higher for participants with 1 or 2 allergic parents, especially in early life and at age 30 years (Figure 1).

### Current AD and Age of Onset

AD onset between ages 3 to 5 years had 63% lower odds of current AD than onset at ages 1 to 2 years. The group with an onset age between ages 6 to 15 years (school-aged children and adolescents) had 78% lower odds of current AD (model 1 in eFigure 3 in Supplement 1). Including early sensitization in the model (model 2 in eFigure 3 in Supplement 1) slightly reduced the odds ratios for later AD onset, but the differences between the 2 models were trivial.

### AD Status in Young Adulthood and Probability of Remission

Among 572 participants with AD onset until the age of 15 years, 497 (87%) experienced remission (ie, absence of AD symptoms) during late adolescence and/or young adulthood. This proportion was slightly higher (12 of 13 [92%]) in the small group of individuals with AD onset during prepuberty and puberty. These frequencies are reported without adjustment for confounders (Table 1).

**Table 1.** AD Status During Late Adolescence and Young Adulthood Among 572 Participants With AD Onset Before Age 15 Years

Potential determinants	Participants by AD status during late adolescence and young adulthood, No. (column %)^a^		Total (n = 572), No. (row %)
	AD symptoms^b^ (n = 75)	No AD symptoms (n = 497)^c^	
Age of AD onset, y			
1-2	56 (13)	359 (87)	415 (100)
3-5	14 (12)	100 (88)	114 (100)
6-10	4 (13)	26 (87)	30 (100)
11-15	1 (8)	12 (92)	13 (100)
Sex			
Female	48 (17)	233 (83)	281 (100)
Male	27 (9)	264 (91)	291 (100)
Parental allergy status^d^			
≥1 Parent	55 (16)	297 (84)	352 (100)
Nonallergic parents	19 (9)	182 (91)	201 (100)
Early allergic sensitization^e^			
Yes	37 (17)	175 (83)	212 (100)
No	6 (8)	71 (92)	77 (100)

Abbreviation: AD, atopic dermatitis.

^a^ AD status was assessed at age 20 years (late adolescence) and age 30 years (young adulthood).

^b^ Includes both persistent symptoms and a relapse after puberty.

^c^ Includes 264 participants who had missing data and no AD symptoms at the 2 follow-up assessments at ages 20 or 30 years.

^d^ Parental allergy status was unknown for 19 participants.

^e^ Sensitization to common allergens during the first 5 years of life; unknown for 283 participants.

**Figure 2.**
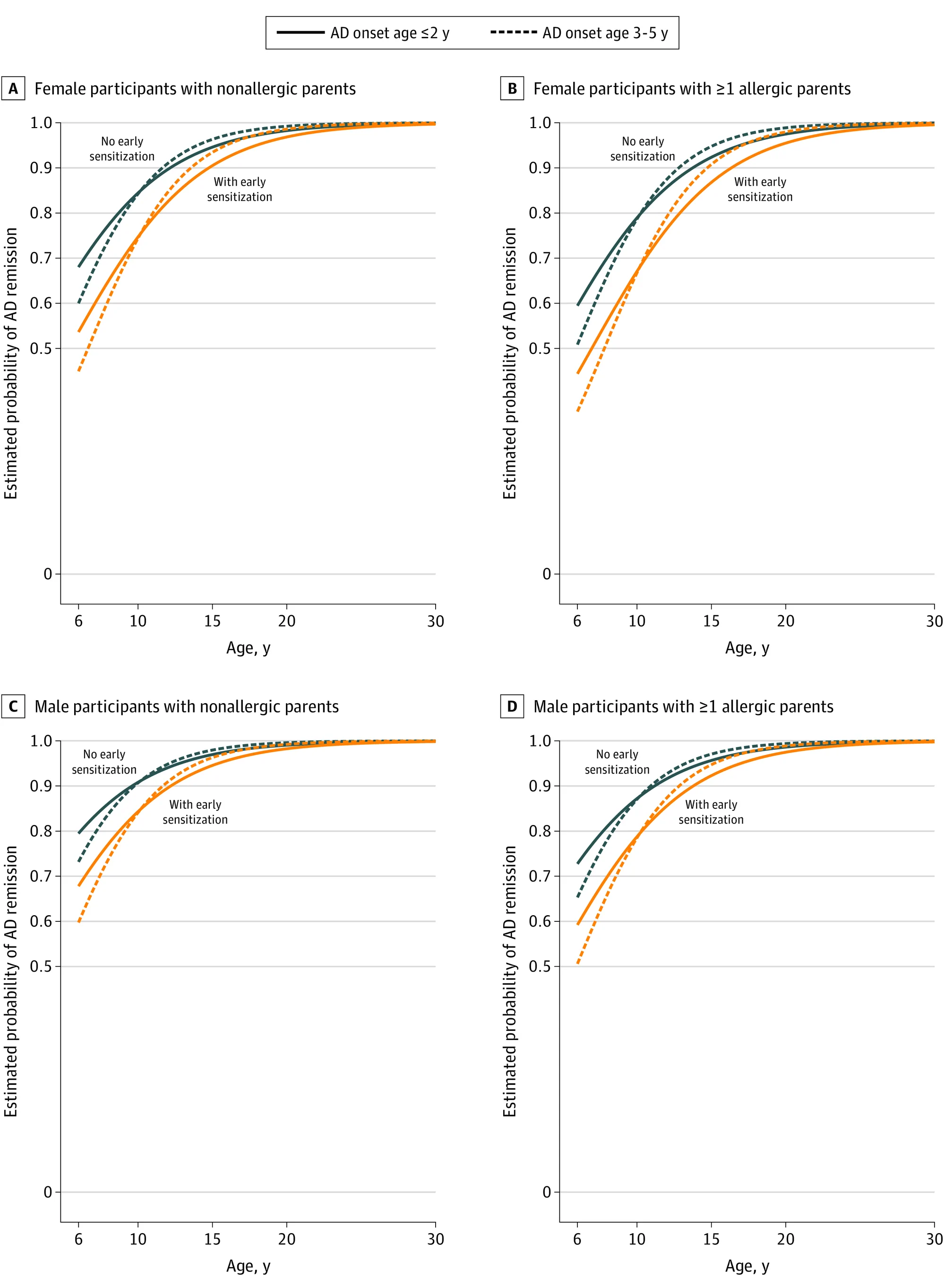
Estimated Probability of Atopic Dermatitis (AD) Remission During Ages 6 to 30 Years for Children With Onset in the First 5 Years of Life Estimated probabilities were presented on the basis of combinations of factors: age of AD early onset (ie, during the first 5 years, dichotomized into 2 groups), sex, parental allergy status, and early (ie, during the first 5 years) allergic sensitization to at least 1 of 7 common aero- or food allergens. AD remission at each follow-up assessment was defined as no AD symptom during the last 12 months and can be observed multiple times. For example, participants with AD onset in the first 2 years of life who were in the highest risk group (ie, female, sensitized early in life, and having at least 1 allergic parent [represented by the solid orange line in panel B]), had an 80% chance to experience remission at age 13 years.

Based on the GLMM model, among 658 children with onset within the first 5 years, male sex and absence of early allergic sensitization were associated with a higher chance of AD remission between ages 6 to 30 years (eTable 5 in Supplement 1). The estimated probability of AD remission (ie, at least 12 months symptom-free) increased with age (Figure 2). Upon further investigation, a consistent crossover across various risk groups was identified at the age of 10 years. Before the age of 10 years, the probability of AD remission was higher for the group with onset until age 2 years. However, after reaching age 10 years, this group displayed a relatively lower chance of remission compared to the group with onset between 3 and 5 years. For example, among male patients with nonallergic parent and no early allergic sensitization with AD onset at or younger than 2 years vs at ages 3 to 5 years, the estimated probabilities of AD remission at age 6 years were 0.79 vs 0.73; at age 10 years, 0.91 vs 0.91; and at age 20 years, 0.99 vs 1.00.

### Remission Trajectories and Early-Life Factors

**Figure 3.**
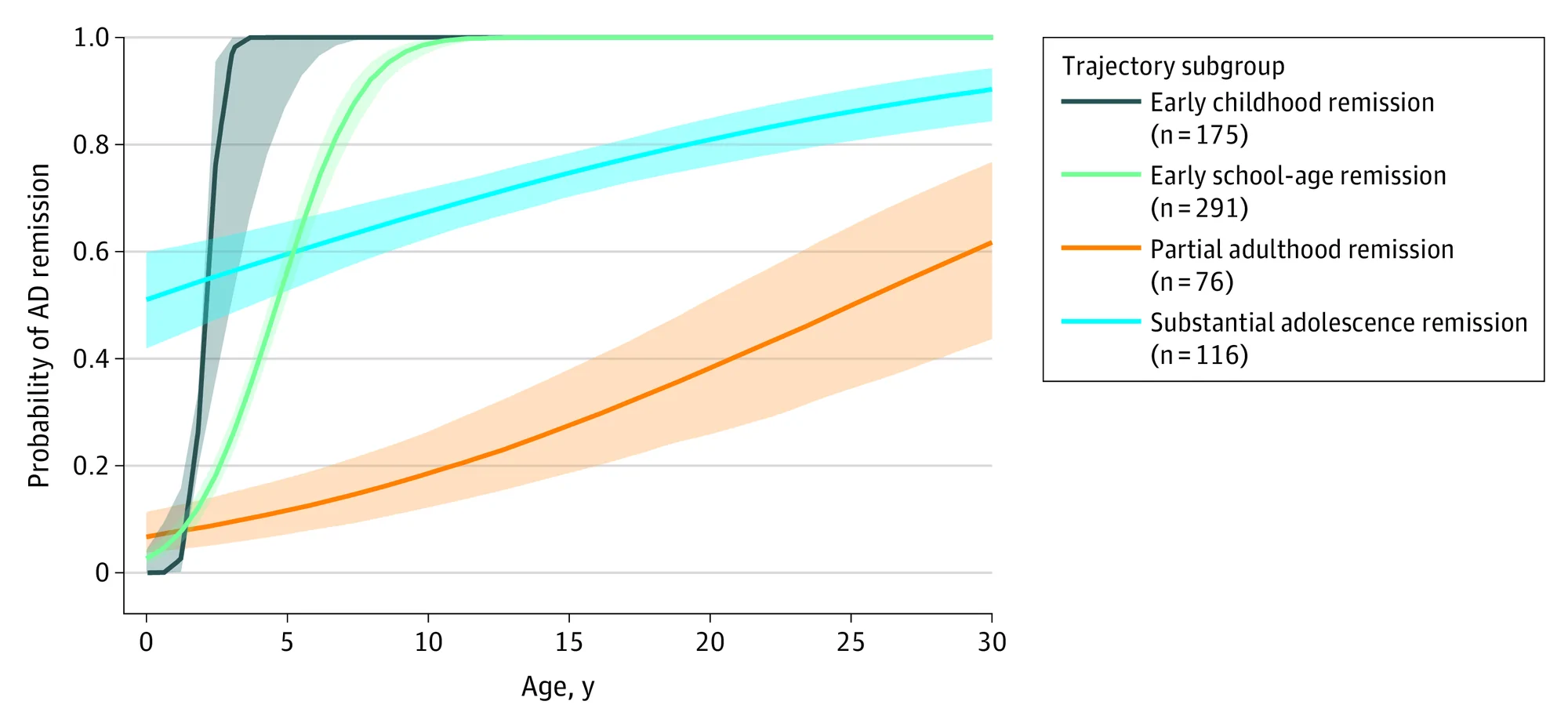
Four Trajectories of Atopic Dermatitis (AD) Remission in the German Multicenter Allergy Study Birth Cohort Participants Who Developed AD in the First 5 Years of Life Subgroups were identified from the 658 participants using growth mixture model. Shaded areas indicate 95% CIs.

Four trajectories of AD remission were identified among 658 children with early-onset AD: (1) early childhood remission (175 [26.6%]); (2) early school-age remission (291 [44.2%]); (3) substantial adolescence remission (116 [17.6%]) with remission probabilities greater than 0.5 across 30 years; and (4) partial adulthood remission (76 [11.6%]), with the lowest probability of AD remission (Figure 3; eTable 6 in Supplement 1). By summarizing partial- and substantial-remission trajectories and early-remission trajectories into 2 major phenotypes, we found that onset at age 2 years or younger, female sex, early allergic sensitization, rhinitis during ages 3 to 5 years, and asthma at age 6 years were associated with a higher risk of being classified into the persistent phenotype (Table 2).

**Table 2.** Association of Early-Life Factors With AD Phenotypes Based on 4 Remission Trajectories for Children With Early-Onset AD in the German MAS Birth Cohort

Early-life factors	No. (%)		RR (95% CI)^a^	
	Persistent phenotype (n = 192)^b^	Remission phenotype (n = 466)^c^	Crude	Adjusted
Onset between 1-2 y (vs onset between 3-5 y)	164 (85.4)	356 (76.4)	1.55 (1.09-2.22)	1.59 (1.12-2.26)
Female sex (vs male sex)	112 (58.3)	196 (42.1)	1.59 (1.25-2.03)	1.56 (1.23-2.00)
≥1 Allergic parents (vs nonallergic parents)	131 (70.1)	271 (61.6)	1.31 (1.00-1.71)	1.28 (0.98-1.66)
Allergic sensitization during first 5 y of life (vs none)	94 (84.7)	142 (67.6)	1.99 (1.27-3.13)	1.95 (1.25-3.05)
Rhinitis at ages 3-5 y (vs none)	74 (39.6)	114 (26.3)	1.51 (1.19-1.91)	1.54 (1.21-1.95)
Asthma at age 6 y (vs none)	17 (9.9)	8 (2.1)	2.31 (1.71-3.12)	2.22 (1.70-2.89)

Abbreviations: AD, atopic dermatitis; RR, risk ratio.

^a^ Risk ratios were estimated using log-binomial regression. For adjusted RR, parental allergy status was adjusted for sex; and sex was adjusted for parental allergy status; all other variables were adjusted for parental allergy status and sex.

^b^ The persistent phenotype consisted of 2 subgroups: substantial adolescence remission and partial adulthood remission.

^c^ As the reference group, the remission phenotype consisted of 2 subgroups: early childhood remission and early school age remission.

## Discussion

In MAS, both AD incidence and prevalence peaked during the first 5 years of life, decreased afterwards, and resurged slightly after early adolescence, extending to age 30 years. AD onset after the age of 2 years had lower odds of current AD. Four remission trajectories were identified. Onset at age 2 years or younger, female sex, early allergic sensitization, rhinitis during ages 3 to 5 years, and asthma at age 6 years were associated with the persistent phenotype.

### AD Incidence and Prevalence

Our results demonstrated an increasing trend of AD for 1-year incidence and 1-year prevalence after late adolescence. Such a potential increase in AD prevalence and incidence after adolescence was reported in a global AD assessment^24^ and in studies from the United Kingdom and Denmark.^6,10,25,26^ Furthermore, we reported an AD prevalence of more than 20% before age 5 years, higher than that what has been reported in other longitudinal studies, with prevalence ranging from 10% to 20%.^27,28,29,30^ In contrast to the incidence of eczema in England (150 per 1000 person-years), where the incidence peaks at year 1,^10^ the AD incidence rate in our MAS cohort was substantially higher (217 and 344 per 1000 person-years among infants without and with allergic parents, respectively). The higher AD prevalence and incidence in early life observed in our cohort could be attributed to the deliberate sampling of a slightly risk-enriched sample, resulting in an elevated incidence rate compared with the general population. Besides, unlike the follow-ups starting from age 8 years onward, which relied on parent- or self-reported questionnaires with lower incidences, our case definition during the initial 7 years of life included study physicians’ assessments and parent-reported symptoms, potentially contributing to the higher AD incidences observed. Moreover, our urban-based cohort might have higher AD incidence and prevalence, as suggested in studies in England and Italy.^10,31^

### Age of Onset as a Risk Factor

The decreased risk for young adult–onset AD after adjusting for early allergic sensitization (ORs changed slightly from 0.30 to 0.13) (eFigure 3 in Supplement 1) underscores that the observed association of AD onset during late adolescence and early adulthood might be explained mostly by early allergic sensitization. Additionally, AD onset during prepuberty and puberty may lead to a milder disease course than onset in early childhood.

Among patients with AD onset during the first 5 years of life, those with onset at age 2 years or younger appeared more likely to achieve remission before age 10 years than those with onset at 3 to 5 years. However, those with a younger age of onset had a lower remission chance afterward than those with onset at 3 to 5 years. Results from log-binomial regression showed an elevated risk of a persistent phenotype for onset at 2 years or younger. Studies on AD phenotypes typically identify early-onset groups with a common cutoff around age 2 years, followed by transient or persistent trajectories.^8,32,33^ Therefore, the association of age of early-onset may vary over time due to heterogeneous subgroups of AD progression and remains inconclusive.

### Potential Factors Associated With Remission Trajectories

Our trajectory analysis suggested that subgroups with different remission times and persistence levels may represent distinct phenotypes of early-onset AD. This finding aligns with latent classes identified in UK and Netherlands cohorts with follow-up to 11 and 16 years.^34^ However, our long-term birth cohort data enables identification of subgroups with more precise remission times, rather than just early or late resolution.

We found that the persistent phenotype with a consistently medium or low chance of AD remission was associated with several factors, including onset at age 2 years or younger, female sex, early sensitization to common allergens, and allergic comorbidity in early life. Similar observations were reported in a Japanese birth cohort, which found an association of respiratory allergic symptoms and IgE sensitization with the persistent eczema phenotype.^35^ Parental allergy status, however, was not relevantly associated with AD remission subgroups. While filaggrin mutation, parental allergy status, and early sensitization to common allergens are known to influence AD persistence^28,32,36^ and remission,^11,37^ other studies have found no association between AD persistence and allergen sensitivities^36^ or family history of allergic disease into adulthood.^25^ These discrepancies may result from differences in variable definitions and follow-up durations. Thus, although parental allergy status was not a relevant factor in our study, it should not be entirely dismissed.^11,38^

### Other Factors

Our data show more incident cases in female than male participants after age 5 years. This is consistent with recent findings from the Swedish birth cohort study BAMSE^9^ and 2 British birth cohorts.^6^ Likewise, among early-onset AD, males had a higher chance of remission, as supported by the BAMSE study with follow-up to 16 years.^37^ Additionally, individuals with AD onset between ages 1 and 2 years, male participants, participants with nonallergic parents, and those with no early allergic sensitization had a higher proportion of loss to follow-up after ages 20 and 30 years. This may indicate that those with milder or asymptomatic AD were less motivated to complete the questionnaire, and the overestimation of AD occurrence might exist (eFigure 2 and eTable 3 in Supplement 1).

### Limitations

MAS, distinguished by long-term follow-up, enables a comprehensive investigation of AD trajectory, but this study has limitations. First, a major challenge in longitudinal AD research is the change in AD definition over time.^9,39^ To capture AD in infancy, we summarized clinical diagnoses and symptoms across shorter follow-up intervals. Furthermore, AD diagnosis—based on clinical assessment or reported symptoms—remained consistent, ensuring our case definition. Second, unmeasured or time-varying covariates may limit interpretation. For instance, early allergic sensitization was available only at certain time points, with some missing data, and was summarized as a binary variable. AD severity^8,36,40^ was not incorporated due to incomplete information, with more than 70% missing for Scoring AD (SCORAD) scores. Similarly, filaggrin gene mutation data were not included, as they were available for only 66% of participants. We prioritized clinically available information to ensure easy verification by epidemiologic studies. Given that filaggrin loss-of-function mutation prevalence varies by ethnicity^41^ and maternal filaggrin mutations may influence AD risk in children,^42^ incorporating parental allergy status should have addressed this genetic factor to some extent. Third, given possible selective attrition (with 30-year follow-up information available for 482 participants), multiple imputation was not implemented; findings should be interpreted cautiously. Furthermore, while this risk-enriched cohort may not fully represent the broader German population, it provides valuable insights for urban areas with higher allergy prevalence.^43,44,45^ Likewise, our findings may not generalize to other ethnic groups,^46,47^ as more than 90% of cohort participants were White.

Further investigation is needed to explore associations between early-life AD and allergic comorbidities (asthma and/or rhinitis). Identifying high-risk groups for early immunomodulating interventions may prevent disease progression and allergic multimorbidity.^14^ Extending birth cohort follow-ups into mid- and late adulthood could enhance understanding of the natural history of AD.^4^

## Conclusions

Our study found that most AD cases first occur before school age in this predominantly White birth cohort study from Germany. Among participants who developed AD during the first 5 years of life, more than 87% experienced remission in adulthood (ie, not having AD symptoms at 20 and 30 years of age). Onset at older than 2 years, male sex, and absence of sensitization against common allergens or allergic respiratory diseases in early childhood were independently associated with higher chances of remission. These findings underscore the association of early-life factors on AD in adulthood and can help parents and health care professionals better manage the course of early childhood AD, potentially improving treatment for individuals at risk of persistence.

## Notes

Supplement 1. **eFigure 1. **Prevalence of Atopic Dermatitis (AD) in German MAS Birth Cohort Participants (All Born in 1990) Who Were Followed Up Over 30 Years **eFigure 2.** Incidence of Atopic Dermatitis (AD) in German MAS Birth Cohort Study **eFigure 3.** Forest Plot of the Association of Current Atopic Dermatitis (AD) and Age of AD Onset, Adjusted for Life Stage, Sex, Parental Allergy Status, and Early Allergic Sensitization (During the First 5 Years of Life) **eTable 1.** Detailed Definition of Atopic Dermatitis (AD) at Different Follow-Up Assessments **eTable 2.** Characteristics of the Birth Cohort Study Population by Atopic Dermatitis (AD) Status **eTable 3.** Atopic Dermatitis (AD) Status During Late Adolescence (Assessed at age 20 Years) and Young Adulthood (Assessed at age 30 Years) by Age of AD Onset, Sex, Parental Allergy Status, and Early Allergic Sensitization, Including Missing at Both Assessments in Adulthood (Age 20 and 30 Years) **eTable 4.** Sensitivity Analysis of the Association of Atopic Dermatitis (AD) Occurrence During Age 1 to 30 Years and Age of AD Onset, Adjusted for Life Stage, Sex, Parental Allergic Status, and Early Allergic Sensitization (During the First 5 Years of Life) **eTable 5.** Association of Atopic Dermatitis (AD) Remission During Age 6 to 30 Years and Age of Early AD Onset Including Interaction Terms With Age Using Generalized Linear Mixed Model **eTable 6.** Early Life Characteristics by Atopic Dermatitis (AD) Remission Subgroups Among Children With Early Onset (ie, AD During the First 5 Years) in German MAS Birth Cohort **eTable 7.** Sensitivity Analysis of Association of Early Life Characteristics and Trajectories of Atopic Dermatitis (AD) Remission for the Children With Early Onset (ie, AD During the First 5 Years) in German MAS Birth Cohort
eMethods 1.

eMethods 2.

eMethods 3.

Supplement 2.
Data Sharing Statement

## References

[1] Weidinger S, Novak N. Atopic dermatitis. Lancet. 2016;387(10023):1109-1122. doi:10.1016/S0140-6736(15)00149-X 26377142

[2] Lee HH, Patel KR, Singam V, Rastogi S, Silverberg JI. A systematic review and meta-analysis of the prevalence and phenotype of adult-onset atopic dermatitis. J Am Acad Dermatol. 2019;80(6):1526-1532.e7. doi:10.1016/j.jaad.2018.05.1241 29864464

[3] Bylund S, Kobyletzki LB, Svalstedt M, Svensson Å. Prevalence and incidence of atopic dermatitis: a systematic review. Acta Derm Venereol. 2020;100(12):adv00160. doi:10.2340/00015555-3510 32412646 PMC9189744

[4] Möhrenschlager M, Seeli C, Anasiewicz N. Atopic dermatitis in a population-based cohort from Stockholm, Sweden 24 years after start: new data, new questions. J Eur Acad Dermatol Venereol. 2022;36(5):634-634. doi:10.1111/jdv.18085 35416366

[5] Margolis JS, Abuabara K, Bilker W, Hoffstad O, Margolis DJ. Persistence of mild to moderate atopic dermatitis. JAMA Dermatol. 2014;150(6):593-600. doi:10.1001/jamadermatol.2013.10271 24696036 PMC4352328

[6] Abuabara K, Ye M, McCulloch CE, . Clinical onset of atopic eczema: results from 2 nationally representative British birth cohorts followed through midlife. J Allergy Clin Immunol. 2019;144(3):710-719. doi:10.1016/j.jaci.2019.05.040 31260715 PMC6721832

[7] Chovatiya R, Silverberg JI. Evaluating the longitudinal course of atopic dermatitis: implications for clinical practice. Am J Clin Dermatol. 2022;23(4):459-468. doi:10.1007/s40257-022-00697-w 35639253 PMC10166131

[8] Wan J, Mitra N, Hoffstad OJ, Yan AC, Margolis DJ. Longitudinal atopic dermatitis control and persistence vary with timing of disease onset in children: a cohort study. J Am Acad Dermatol. 2019;81(6):1292-1299. doi:10.1016/j.jaad.2019.05.016 31085263 PMC6892595

[9] Johansson EK, Bergström A, Kull I, . Prevalence and characteristics of atopic dermatitis among young adult females and males-report from the Swedish population-based study BAMSE. J Eur Acad Dermatol Venereol. 2022;36(5):698-704. doi:10.1111/jdv.17929 35032357 PMC9303811

[10] de Lusignan S, Alexander H, Broderick C, . The epidemiology of eczema in children and adults in England: a population-based study using primary care data. Clin Exp Allergy. 2021;51(3):471-482. doi:10.1111/cea.13784 33179341 PMC7984097

[11] Illi S, von Mutius E, Lau S, ; Multicenter Allergy Study Group. The natural course of atopic dermatitis from birth to age 7 years and the association with asthma. J Allergy Clin Immunol. 2004;113(5):925-931. doi:10.1016/j.jaci.2004.01.778 15131576

[12] Gough H, Grabenhenrich L, Reich A, ; MAS study group. Allergic multimorbidity of asthma, rhinitis and eczema over 20 years in the German birth cohort MAS. Pediatr Allergy Immunol. 2015;26(5):431-437. doi:10.1111/pai.12410 26011739 PMC4744942

[13] Lau S, Matricardi PM, Wahn U, Lee YA, Keil T. Allergy and atopy from infancy to adulthood: messages from the German birth cohort MAS. Ann Allergy Asthma Immunol. 2019;122(1):25-32. doi:10.1016/j.anai.2018.05.012 29803707

[14] Spergel JM, Du Toit G, Davis CM. Might biologics serve to interrupt the atopic march? J Allergy Clin Immunol. 2023;151(3):590-594. doi:10.1016/j.jaci.2023.01.001 36681581

[15] Bergmann RL, Bergmann KE, Lau-Schadensdorf S, et al. Atopic diseases in infancy: the German multicenter atopy study (MAS-90). Pediatr Allergy Immunol. 1994;5(6 Suppl):19-25. doi:10.1111/j.1399-3038.1994.tb00343.x 7728224

[16] Grabenhenrich LB, Gough H, Reich A, et al. Early-life determinants of asthma from birth to age 20 years: a German birth cohort study. J Allergy Clin Immunol. 2014;133(4):979-988. doi:10.1016/j.jaci.2013.11.035 24461583

[17] Asher MI, Keil U, Anderson HR, . International Study of Asthma and Allergies in Childhood (ISAAC): rationale and methods. Eur Respir J. 1995;8(3):483-491. doi:10.1183/09031936.95.08030483 7789502

[18] Herle M, Micali N, Abdulkadir M, . Identifying typical trajectories in longitudinal data: modelling strategies and interpretations. Eur J Epidemiol. 2020;35(3):205-222. doi:10.1007/s10654-020-00615-6 32140937 PMC7154024

[19] Nguena Nguefack HL, Pagé MG, Katz J, . Trajectory modelling techniques useful to epidemiological research: a comparative narrative review of approaches. Clin Epidemiol. 2020;12:1205-1222. doi:10.2147/CLEP.S265287 33154677 PMC7608582

[20] Flores NM, Lovinsky-Desir S, Divjan A, . Trajectory analysis of rhinitis in a birth cohort from lower-income New York City neighborhoods. J Allergy Clin Immunol. 2024;154(1):111-119. doi:10.1016/j.jaci.2023.11.919 38104949 PMC11180217

[21] Proust-Lima C, Philipps V, Liquet B. Estimation of extended mixed models using latent classes and latent processes: the R package lcmm. J Stat Softw. 2017;78(2):1-56. doi:10.18637/jss.v078.i02

[22] Proust-Lima C, Philipps V, Diakite A, Liquet B. lcmm: Extended mixed models using latent classes and latent processes. Accessed October 15, 2025. https://cran.r-project.org/package=lcmm

[23] Grabenhenrich LB, Gough H, Reich A, . Early-life determinants of asthma from birth to age 20 years: a German birth cohort study. J Allergy Clin Immunol. 2014;133(4):979-988. doi:10.1016/j.jaci.2013.11.035 24461583

[24] Laughter MR, Maymone MBC, Mashayekhi S, . The global burden of atopic dermatitis: lessons from the Global Burden of Disease Study 1990-2017. Br J Dermatol. 2021;184(2):304-309. doi:10.1111/bjd.19580 33006135

[25] Mortz CG, Andersen KE, Dellgren C, Barington T, Bindslev-Jensen C. Atopic dermatitis from adolescence to adulthood in the TOACS cohort: prevalence, persistence and comorbidities. Allergy. 2015;70(7):836-845. doi:10.1111/all.12619 25832131

[26] Abuabara K, Langan SM. Atopic dermatitis across the life course. Br J Dermatol. 2023;188(6):709-717. doi:10.1093/bjd/ljac072 36715326

[27] Abuabara K, Yu AM, Okhovat JP, Allen IE, Langan SM. The prevalence of atopic dermatitis beyond childhood: a systematic review and meta-analysis of longitudinal studies. Allergy. 2018;73(3):696-704. doi:10.1111/all.13320 28960336 PMC5830308

[28] Ballardini N, Kull I, Lind T, . Development and comorbidity of eczema, asthma and rhinitis to age 12: data from the BAMSE birth cohort. Allergy. 2012;67(4):537-544. doi:10.1111/j.1398-9995.2012.02786.x 22335548

[29] Ziyab AH, Raza A, Karmaus W, . Trends in eczema in the first 18 years of life: results from the Isle of Wight 1989 birth cohort study. Clin Exp Allergy. 2010;40(12):1776-1784. doi:10.1111/j.1365-2222.2010.03633.x 21059120

[30] Nissen SP, Kjaer HF, Høst A, Nielsen J, Halken S. The natural course of sensitization and allergic diseases from childhood to adulthood. Pediatr Allergy Immunol. 2013;24(6):549-555. doi:10.1111/pai.12108 23902477

[31] Pesce G, Marcon A, Carosso A, . Adult eczema in Italy: prevalence and associations with environmental factors. J Eur Acad Dermatol Venereol. 2015;29(6):1180-1187. doi:10.1111/jdv.12784 25363318

[32] Roduit C, Frei R, Depner M, ; the PASTURE study group. Phenotypes of atopic dermatitis depending on the timing of onset and progression in childhood. JAMA Pediatr. 2017;171(7):655-662. doi:10.1001/jamapediatrics.2017.0556 28531273 PMC5710337

[33] Li H, Dai T, Liu C, Liu Q, Tan C. Phenotypes of atopic dermatitis and the risk for subsequent asthma: a systematic review and meta-analysis. J Am Acad Dermatol. 2022;86(2):365-372. doi:10.1016/j.jaad.2021.07.064 34384834

[34] Paternoster L, Savenije OEM, Heron J, . Identification of atopic dermatitis subgroups in children from 2 longitudinal birth cohorts. J Allergy Clin Immunol. 2018;141(3):964-971. doi:10.1016/j.jaci.2017.09.044 29129583 PMC5840507

[35] Kiguchi T, Yamamoto-Hanada K, Saito-Abe M, Fukuie T, Ohya Y. Eczema phenotypes and IgE component sensitization in adolescents: a population-based birth cohort. Allergol Int. 2023;72(1):107-115. doi:10.1016/j.alit.2022.05.012 35781407

[36] Kim JP, Chao LX, Simpson EL, Silverberg JI. Persistence of atopic dermatitis (AD): a systematic review and meta-analysis. J Am Acad Dermatol. 2016;75(4):681-687.e11. doi:10.1016/j.jaad.2016.05.028 27544489 PMC5216177

[37] Johansson EK, Bergström A, Kull I, . Prognosis of preschool eczema and factors of importance for remission. Acta Derm Venereol. 2018;98(7):630-635. doi:10.2340/00015555-2919 29507996

[38] Irvine AD, Mina-Osorio P. Disease trajectories in childhood atopic dermatitis: an update and practitioner’s guide. Br J Dermatol. 2019;181(5):895-906. doi:10.1111/bjd.17766 30758843 PMC6899789

[39] Nakamura T, Haider S, Fontanella S, Murray CS, Simpson A, Custovic A. Modelling trajectories of parentally reported and physician-confirmed atopic dermatitis in a birth cohort study. Br J Dermatol. 2022;186(2):274-284. doi:10.1111/bjd.20767 34564850

[40] Mulick AR, Mansfield KE, Silverwood RJ, . Four childhood atopic dermatitis subtypes identified from trajectory and severity of disease and internally validated in a large UK birth cohort. Br J Dermatol. 2021;185(3):526-536. doi:10.1111/bjd.19885 33655501 PMC8410876

[41] Gupta J, Margolis DJ. Filaggrin gene mutations with special reference to atopic dermatitis. Curr Treat Options Allergy. 2020;7(3):403-413. doi:10.1007/s40521-020-00271-x 33585163 PMC7880084

[42] Esparza-Gordillo J, Matanovic A, Marenholz I, . Maternal filaggrin mutations increase the risk of atopic dermatitis in children: an effect independent of mutation inheritance. PLoS Genet. 2015;11(3):e1005076. doi:10.1371/journal.pgen.1005076 25757221 PMC4355615

[43] Tizek L, Redlinger E, Ring J, Eyerich K, Biedermann T, Zink A. Urban vs rural—prevalence of self-reported allergies in various occupational and regional settings. World Allergy Organ J. 2022;15(1):100625. doi:10.1016/j.waojou.2022.100625 35145605 PMC8802121

[44] Desalu OO, Adeoti AO, Ojuawo OB, . Urban-rural differences in the epidemiology of asthma and allergies in Nigeria: a population-based study. J Asthma Allergy. 2021;14:1389-1397. doi:10.2147/JAA.S333133 34866916 PMC8637762

[45] Ricciardo BM, Kessaris HL, Kumarasinghe P, Carapetis JR, Bowen AC. The burden of atopic dermatitis and bacterial skin infections among urban-living Indigenous children and young people in high-income countries: a systematic review. Pediatr Dermatol. 2023;40(1):35-43. doi:10.1111/pde.15153 36349531 PMC10946708

[46] Kim Y, Blomberg M, Rifas-Shiman SL, . Racial/ethnic differences in incidence and persistence of childhood atopic dermatitis. J Invest Dermatol. 2019;139(4):827-834. doi:10.1016/j.jid.2018.10.029 30414911 PMC6431568

[47] Brunner PM, Guttman-Yassky E. Racial differences in atopic dermatitis. Ann Allergy Asthma Immunol. 2019;122(5):449-455. doi:10.1016/j.anai.2018.11.015 30465859

