# Supplementary material for: Incidence and Remission of Atopic Dermatitis in a German Birth Cohort
Source: JAMA Netw Open. 2025 Dec 8;8(12):e2544324. doi: 10.1001/jamanetworkopen.2025.44324 (PMC12687101; doi:10.1001/jamanetworkopen.2025.44324)
Supplement: Supplement 1. — eFigure 1. Prevalence of Atopic Dermatitis (AD) in German MAS Birth Cohort Participants (All Born in 1990) Who Were Followed Up Over 30 Years eFigure 2. Incidence of Atopic Dermatitis (AD) in German MAS Birth Cohort Study eFigure 3. Forest Plot of the Association of Current Atopic Dermatitis (AD) and Age of AD Onset, Adjusted for Life Stage, Sex, Parental Allergy Status, and Early Allergic Sensitization (During the First 5 Years of Life) eTable 1. Detailed Definition of Atopic Dermatitis (AD) at Different Follow-Up Assessments eTable 2. Characteristics of the Birth Cohort Study Population by Atopic Dermatitis (AD) Status eTable 3. Atopic Dermatitis (AD) Status During Late Adolescence (Assessed at age 20 Years) and Young Adulthood (Assessed at age 30 Years) by Age of AD Onset, Sex, Parental Allergy Status, and Early Allergic Sensitization, Including Missing at Both Assessments in Adulthood (Age 20 and 30 Years) eTable 4. Sensitivity Analysis of the Association of Atopic Dermatitis (AD) Occurrence During Age 1 to 30 Years and Age of AD Onset, Adjusted for Life Stage, Sex, Parental Allergic Status, and Early Allergic Sensitization (During the First 5 Years of Life) eTable 5. Association of Atopic Dermatitis (AD) Remission During Age 6 to 30 Years and Age of Early AD Onset Including Interaction Terms With Age Using Generalized Linear Mixed Model eTable 6. Early Life Characteristics by Atopic Dermatitis (AD) Remission Subgroups Among Children With Early Onset (ie, AD During the First 5 Years) in German MAS Birth Cohort eTable 7. Sensitivity Analysis of Association of Early Life Characteristics and Trajectories of Atopic Dermatitis (AD) Remission for the Children With Early Onset (ie, AD During the First 5 Years) in German MAS Birth Cohort eMethods 1. eMethods 2. eMethods 3. [file jamanetwopen-e2544324-s001.pdf]

Supplemental Online Content

Hung CW, Roll S, Icke K, et al. Incidence and remission of atopic dermatitis in a German birth cohort. *JAMA Netw Open*. 2025;8(11):e2544324.
doi:10.1001/jamanetworkopen.2025.44324

eFigure 1. Prevalence of Atopic Dermatitis (AD) in German MAS Birth Cohort Participants (All Born in 1990) Who Were Followed Up Over 30 Years

eFigure 2. Incidence of Atopic Dermatitis (AD) in German MAS Birth Cohort Study

eFigure 3. Forest Plot of the Association of Current Atopic Dermatitis (AD) and Age of AD Onset, Adjusted for Life Stage, Sex, Parental Allergy Status, and Early Allergic Sensitization (During the First 5 Years of Life)

eTable 1. Detailed Definition of Atopic Dermatitis (AD) at Different Follow-Up Assessments

eTable 2. Characteristics of the Birth Cohort Study Population by Atopic Dermatitis (AD) Status

eTable 3. Atopic Dermatitis (AD) Status During Late Adolescence (Assessed at age 20 Years) and Young Adulthood (Assessed at age 30 Years) by Age of AD Onset, Sex, Parental Allergy Status, and Early Allergic Sensitization, Including Missing at Both Assessments in Adulthood (Age 20 and 30 Years)

eTable 4. Sensitivity Analysis of the Association of Atopic Dermatitis (AD) Occurrence During Age 1 to 30 Years and Age of AD Onset, Adjusted for Life Stage, Sex, Parental Allergic Status, and Early Allergic Sensitization (During the First 5 Years of Life)

eTable 5. Association of Atopic Dermatitis (AD) Remission During Age 6 to 30 Years and Age of Early AD Onset Including Interaction Terms With Age Using Generalized Linear Mixed Model

eTable 6. Early Life Characteristics by Atopic Dermatitis (AD) Remission Subgroups Among Children With Early Onset (ie, AD During the First 5 Years) in German MAS Birth Cohort

eTable 7. Sensitivity Analysis of Association of Early Life Characteristics and Trajectories of Atopic Dermatitis (AD) Remission for the Children With Early Onset (ie, AD During the First 5 Years) in German MAS Birth Cohort

eMethods 1.

eMethods 2.

eMethods 3.

Commented [JP1]: eTable 6 is not cited in the main text—please add a citation to it.

This supplemental material has been provided by the authors to give readers additional information about their work.

© 2025 Hung CW et al. *JAMA Network Open*.

eFigure 1. Prevalence of Atopic Dermatitis (AD) in German MAS Birth Cohort Participants (All Born in 1990) Who Were Followed Up Over 30 Years

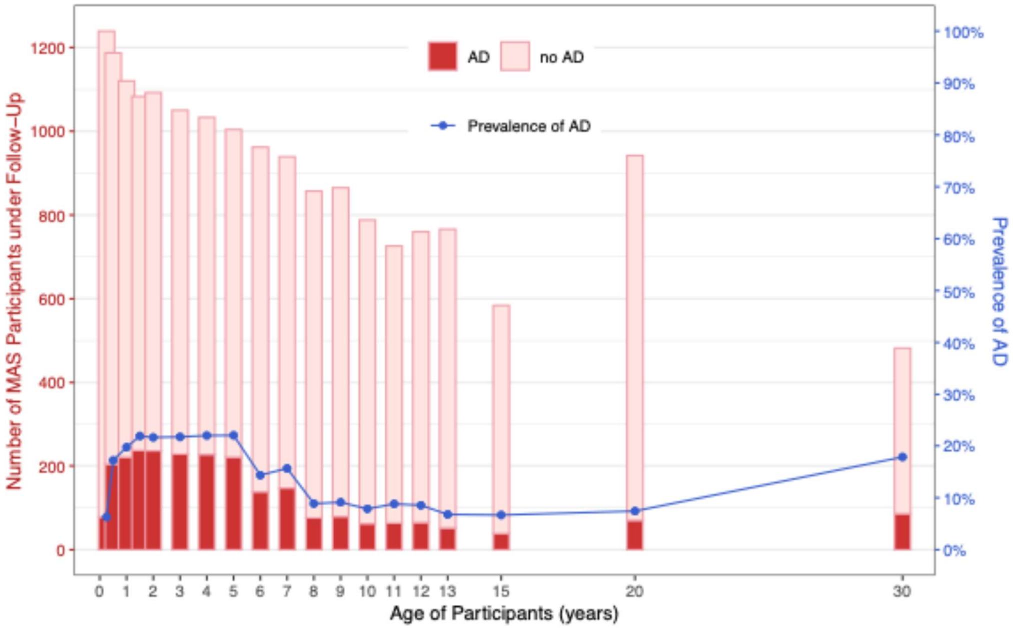

Each bar represents the number of participants who responded at each follow-up. AD cases are marked as dark red and subjects who reported no AD marked as light red (numbers on left y-axis). The blue dots and line represent the prevalence of AD (in %) at each follow-up assessment (percentages on blue right y-axis).

© 2025 Hung CW et al. *JAMA Network Open*.

eFigure 2. Incidence of Atopic Dermatitis (AD) in German MAS Birth Cohort Study

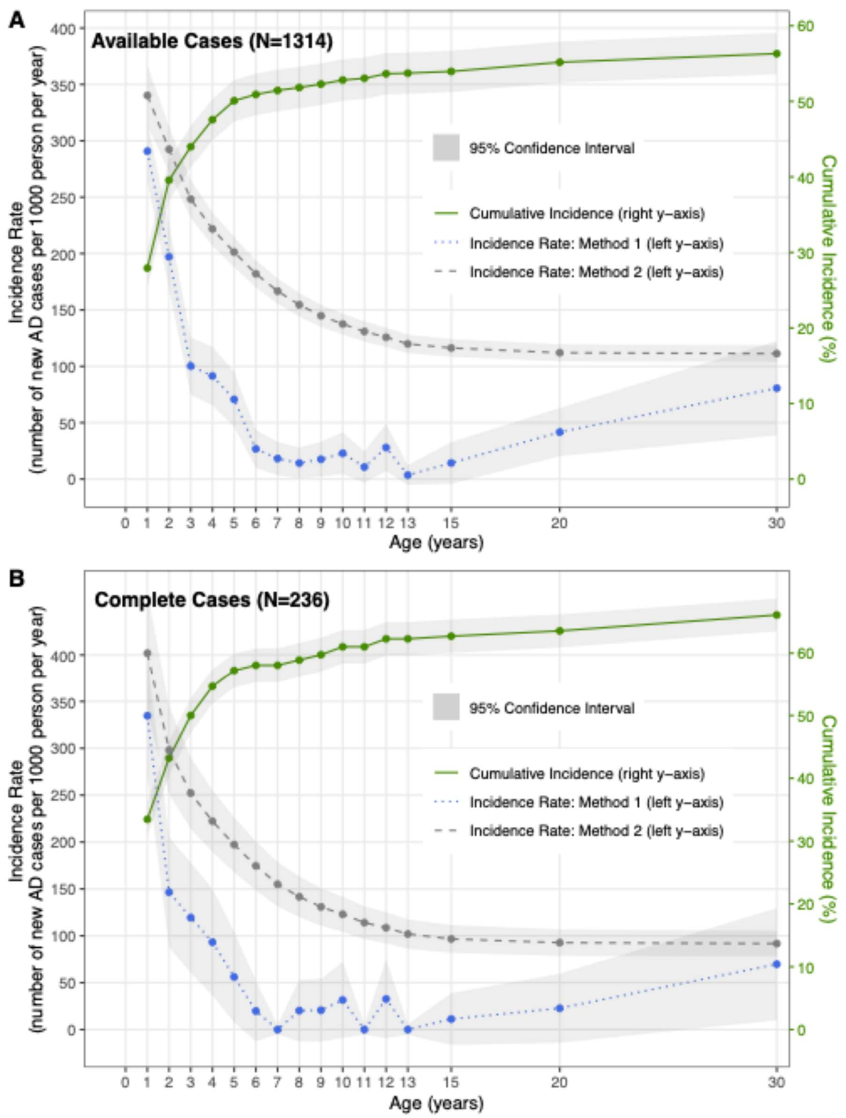

Cumulative incidence (green solid line) used 1,314 subjects (A) and 236 subjects (B) recruited at birth as the denominator for all time points. Incidence rates were calculated using two different methods: i) new cases from the year of the follow-up age (past 12 months) divided by population at risk at respective follow-up age (blue dotted line); ii) cumulative new cases from age one up to the respective follow-up divided by person-years contributed during the observation period (orange dashed line). Every new case was considered to contribute 0.5 years at the respective age. Both incidence rates can be interpreted as the number of new AD cases per 1,000 persons per year. Note: Method ii) assumed that the probability of disease during the observation period from age 1 year to the follow-up age is constant.

© 2025 Hung CW et al. *JAMA Network Open*.

eFigure 3. Forest Plot of the Association of Current Atopic Dermatitis (AD) and Age of AD Onset, Adjusted for Life Stage, Sex, Parental Allergy Status, and Early Allergic Sensitization (During the First 5 Years of Life)

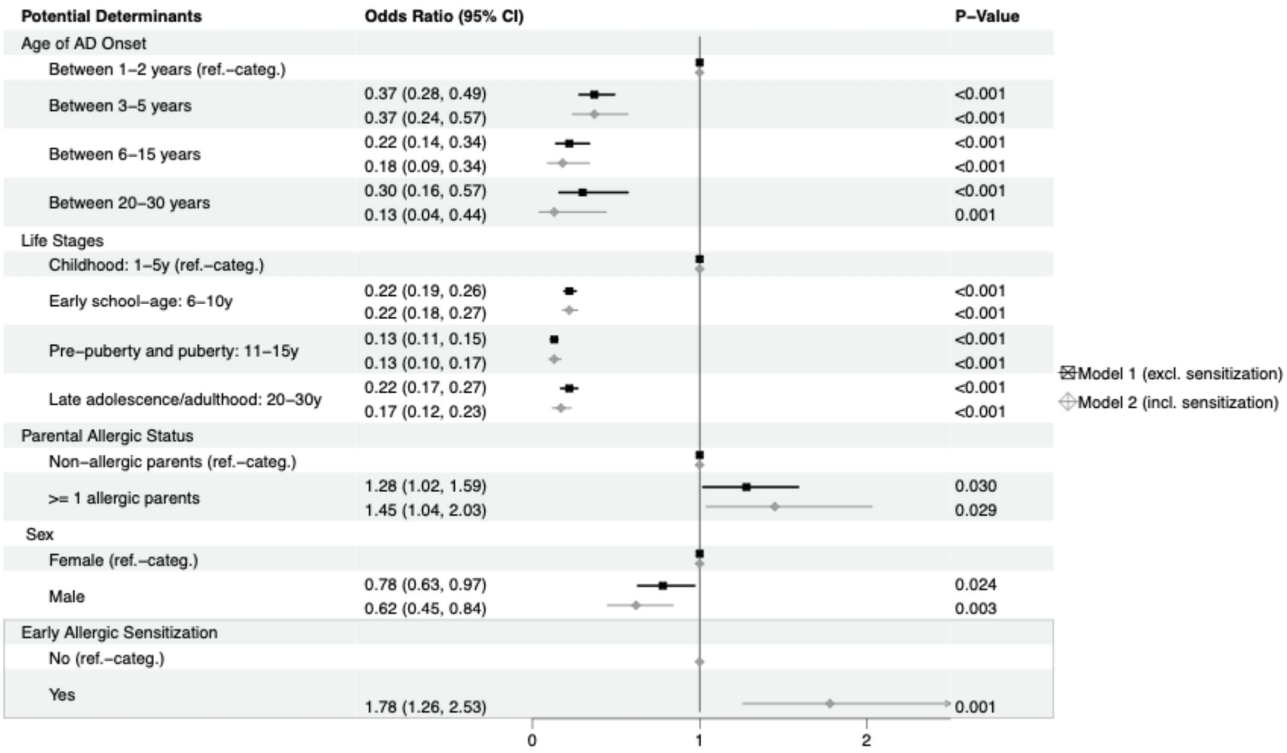

© 2025 Hung CW et al. *JAMA Network Open*.

eTable 1. Detailed Definition of Atopic Dermatitis (AD) at Different Follow-Up Assessments

**Age at Follow-Up**	**AD Definition**
3 months	study doctor assessment
6 months - 2 years	study doctor assessment **and/or** parent-reported symptoms (at least 3 out of the following 4 relevant symptoms – dry skin, scratch, cheek eczema, eczema at other sites [not specified]) after last follow-up or within the last 12 months
3 years	study doctor assessment **and/or** parent-reported symptoms (yes to 3 relevant typical symptoms – dry skin, scratch, eczema within the last 12 months)
4 years	study doctor assessment **and/or** parent-reported symptoms (at least 3 out of the following 4 relevant symptoms – dry skin, scratch, eczema within the last 12 months, eczema at typically affected areas[a])
5 years	study doctor assessment **and/or** parent-reported symptoms (at least 3 out of the following 4 relevant symptoms – dry skin, scratch, itchy rash within the last 12 months, eczema at typically affected areas[a])
6 - 7 years	study doctor assessment **and/or** parent-reported symptoms (itchy rash at typically affected areas[a] within the last 12 months)
8 - 15 years	parent-reported symptoms (itchy rash at typically affected areas[a] within the last 12 months)
20 years	self-reported symptoms (itchy rash at typically affected areas[a] within the last 12 months)
30 years	self-reported symptoms (itchy rash at typically affected areas[a] within the last 12 months) or disease history (doctor diagnosed ever) when itchy rash occurred at other areas

[a]folds of the elbow, behind the knee, at the wrist or the ankle, the face, around the neck, ears, or eyes

© 2025 Hung CW et al. *JAMA Network Open*.

eTable 2. Characteristics of the Birth Cohort Study Population by Atopic Dermatitis (AD) Status

Characteristic	N	Total<br><br>N = 1,314<br>n (%)	Early-onset AD<br>(age ≤5y)<br>N = 658<br>n (%)	Late-onset AD<br>(age >5y)<br>N = 82<br>n (%)	No AD[a]<br><br>N = 574<br>n (%)
**Sex**	1,314				
Female		630 (48%)	308 (47%)	48 (59%)	274 (48%)
Male		684 (52%)	350 (53%)	34 (41%)	300 (52%)
**Parental Allergy Status**	1,236				
One or two allergic parents		707 (57%)	402 (64%)	44 (56%)	261 (49%)
Non-allergic parents		529 (43%)	225 (36%)	35 (44%)	269 (51%)
Unknown		78	31	3	44
**Early Allergic Sensitization**[b]	515				
Yes		345 (67%)	236 (74%)	20 (62%)	89 (55%)
No		170 (33%)	85 (26%)	12 (38%)	73 (45%)
Unknown		799	337	50	412
**Numbers of Follow-Up Available**	1,314				
Median (25th, 75th quartile)		16 (9, 18)	17 (13, 18)	17 (14, 19)	13 (3, 18)
Mean (standard deviation)		13 (6)	15 (4)	15 (5)	11 (7)

[a]For each time point, participants who responded to the questionnaire with incomplete AD information were considered to not have AD. For the whole study period (30 years), those who reported no AD at some follow-up time points while lost to follow-up at partial time points were also counted in this group. There were 27 subjects without responses for all follow-ups.
[b]Immunoglobulin E levels ≥ 0.35 IU/mL against any of 5 common aeroallergens (house dust mite, cat, dog, birch and grass pollen) or 2 common food allergens (cow's milk and hen's egg) during the first 5 years of life.

© 2025 Hung CW et al. *JAMA Network Open*.

eTable 3. Atopic Dermatitis (AD) Status During Late Adolescence (Assessed at age 20 Years) and Young Adulthood (Assessed at age 30 Years) by Age of AD Onset, Sex, Parental Allergy Status, and Early Allergic Sensitization, Including Missing at Both Assessments in Adulthood (Age 20 and 30 Years)

Potential Determinants	AD during late adolescence and young adulthood				Missing[b]		Overall
	AD symptoms (either persistent or a relapse after puberty age)		No AD symptoms (remission at age 20 and 30 years)[a]				
	N = 75<br>n (%)	95% CI	N = 497<br>n (%)	95% CI	N = 137<br>n (%)	95% CI	N = 709<br>n (%)
**Age of AD Onset**							
Between 1-2 years	56 (11%)	8%, 14%	359 (69%)	65%, 73%	105 (20%)	17%, 24%	520 (100%)
Between 3-5 years	14 (10%)	6%, 17%	100 (72%)	64%, 80%	24 (17%)	12%, 25%	138 (100%)
Between 6-10 years	4 (11%)	4%, 27%	26 (72%)	55%, 85%	6 (17%)	7%, 33%	36 (100%)
Between 11-15 years	1 (7%)	0%, 34%	12 (80%)	51%, 95%	2 (13%)	2%, 42%	15 (100%)
**Sex**							
Female	48 (14%)	11%, 19%	233 (69%)	64%, 74%	55 (16%)	13%, 21%	336 (100%)
Male	27 (7%)	5%, 10%	264 (71%)	66%, 75%	82 (22%)	18%, 27%	373 (100%)
**Parental Allergy Status[c]**							
One or two allergic parents	55 (13%)	10%, 16%	297 (69%)	65%, 74%	77 (18%)	15%, 22%	429 (100%)
Non-allergic parents	19 (8%)	5%, 12%	182 (73%)	67%, 79%	47 (19%)	14%, 25%	248 (100%)
**Early Allergic Sensitization[d]**							
Yes	37 (15%)	11%, 20%	175 (69%)	63%, 75%	41 (16%)	12%, 21%	253 (100%)
No	6 (6%)	3%, 14%	71 (76%)	66%, 84%	16 (17%)	10%, 27%	93 (100%)

[a]Including either age 20 or 30 years missing while another time point without AD symptoms
[b]Referring to missing at both ages 20 and 30 years
[c]Unknown (n = 32 subjects)
[d]Sensitization to common allergens during the first 5 years of life; Unknown (n = 363 subjects).
Explanatory note: Among 253 participants with AD and sensitization to common allergens within the first 5 years, 15% (n=37) still had AD in adulthood. In contrast, among the 93 participants with AD and no allergic sensitization, only 6% (n=6) had AD in adulthood. Abbreviations: CI, confidence interval.

eTable 4. Sensitivity Analysis of the Association of Atopic Dermatitis (AD) Occurrence During Age 1 to 30 Years and Age of AD Onset, Adjusted for Life Stage, Sex, Parental Allergic Status, and Early Allergic Sensitization (During the First 5 Years of Life)

	AD – Model 1					
	Available Cases (N=706)			Complete Cases (N=627)		
Predictors	***OR***	***95% CI***	***p-values***	***OR***	***95% CI***	***p-values***
**Age of AD Onset** (vs 1-2 years as ref.-category)						
Between ages 3-5 years	0.37	0.28 – 0.49	<0.001	0.35	0.26 – 0.48	<0.001
Between ages 6-15 years	0.22	0.14 – 0.34	<0.001			
Between ages 20-30 years	0.30	0.16 – 0.57	<0.001			
**Life Stage** (vs first 5 years as ref.-category)						
Early school-age (6-10 years)	0.22	0.19 – 0.26	<0.001	0.17	0.14 – 0.19	<0.001
Pre-puberty and puberty (11-15 years)	0.13	0.11 – 0.15	<0.001	0.09	0.07 – 0.11	<0.001
Late adolescence and early adulthood (20-30 years)	0.22	0.17 – 0.27	<0.001	0.12	0.10 – 0.16	<0.001
**Having 1 or 2 Allergic Parents** (vs non-allergic parents)	1.28	1.02 – 1.59	0.030	1.34	1.04 – 1.73	0.024
**Male Sex** (vs female)	0.78	0.63 – 0.97	0.024	0.75	0.59 – 0.95	0.019
**Early Allergic Sensitization**[a] (vs none)	NA	NA	NA	NA	NA	NA
**Random Effects**[b]						
σ^2	3.29			3.29		
$\tau_{00\ ID}$	1.30_{ID}			1.62_{ID}		
ICC	0.28			0.33		
N_{ID}	706_{ID}			627_{ID}		
Observations	8,692			7,727		
Marginal R^2 / Conditional R^2	0.183 / 0.414			0.200 / 0.465		

[a]Sensitization to at least one of 7 common allergens during the first 5 years of life. [b]Random effects accounted for the variability and difference between study subjects. Abbreviations: OR, odds ratio. CI, confidence interval. NA, not available. σ^2, residual variance under logistic distribution. $\tau_{00\ ID}$, random intercept variance or between-subject variance. ICC, intraclass-correlation coefficient. Marginal R^2 considered only the variance of the fixed effects, while Conditional R^2 considered both the fixed and random effects.

© 2025 Hung CW et al. *JAMA Network Open*.

eTable 4. (continued) Sensitivity Analysis of the Association of Atopic Dermatitis (AD) Occurrence During Age 1 to 30 Years and Age of AD Onset, Adjusted for Life Stage, Sex, Parental Allergic Status, and Early Allergic Sensitization (During the First 5 Years of Life)

	AD – Model 2 (also adjusted for early sensitization)					
	Available Cases (N=340)			Complete Cases (N=308)		
Predictors	***OR***	***95% CI***	***p-values***	***OR***	***95% CI***	***p-values***
**Age of AD Onset** (vs 1-2 years as ref.-category)						
Between ages 3-5 years	0.37	0.24 – 0.57	<0.001	0.35	0.22 – 0.56	<0.001
Between ages 6-15 years	0.18	0.09 – 0.34	<0.001			
Between ages 20-30 years	0.13	0.04 – 0.44	0.001			
**Life Stage** (vs first 5 years as ref.-category)						
Early school-age (6-10 years)	0.22	0.18 – 0.27	<0.001	0.17	0.14 – 0.22	<0.001
Pre-puberty and puberty (11-15 years)	0.13	0.10 – 0.17	<0.001	0.09	0.07 – 0.12	<0.001
Late adolescence and early adulthood (20-30 years)	0.17	0.12 – 0.23	<0.001	0.11	0.08 – 0.16	<0.001
**Having 1 or 2 Allergic Parents** (vs non-allergic parents)	1.45	1.04 – 2.03	0.029	1.49	1.02 – 2.17	0.039
**Male Sex** (vs female)	0.62	0.45 – 0.84	0.003	0.57	0.40 – 0.81	0.002
**Early Allergic Sensitization**[a] (vs none)	1.78	1.26 – 2.53	0.001	1.90	1.28 – 2.83	0.002
**Random Effects**[b]						
σ^2	3.29			3.29		
$\tau_{00\ ID}$	1.45_{ID}			1.76_{ID}		
ICC	0.31			0.35		
N_{ID}	340_{ID}			308_{ID}		
Observations	4,485			4,054		
Marginal R^2 / Conditional R^2	0.210 / 0.451			0.212 / 0.487		

[a]Sensitization to at least one of 7 common allergens during the first 5 years of life. [b]Random effects accounted for the variability and difference between study subjects. Abbreviations: OR, odds ratio. CI, confidence interval. NA, not available. σ^2, residual variance under logistic distribution. $\tau_{00\ ID}$, random intercept variance or between-subject variance. ICC, intraclass-correlation coefficient. Marginal R^2 considered only the variance of the fixed effects, while Conditional R^2 considered both the fixed and random effects.

© 2025 Hung CW et al. *JAMA Network Open*.

eTable 5. Association of Atopic Dermatitis (AD) Remission During Age 6 to 30 Years and Age of Early AD Onset Including Interaction Terms With Age Using Generalized Linear Mixed Model

	**Remission of AD**[a]			
Effect	***Estimate***[b]	**Standard Error**	**z Value**	**Pr > |z|**
(Intercept)	-0.650	0.253	-2.574	0.010
Age of Early AD Onset[c]				
Between 3-5 years	-0.851	0.295	-2.884	0.004
Age	0.234	0.012	19.632	<.0001
Age*Age of Early AD Onset[d]				
age*between 3-5 years	0.084	0.029	2.848	0.004
Having at least one allergic parent (vs both non-allergic)	-0.371	0.196	-1.891	0.059
Male Sex (vs female)	0.599	0.185	3.231	0.001
Early Allergic Sensitization (vs none) in first 5 years[e]	-0.609	0.206	-2.954	0.003

[a]Recurrent event
[b]Results were based on analysis using glmer function in the lme4 package in R. Complete cases (n=308) with 4054 observations were used. Estimates presented above were used to calculate the probability of AD remission in Figure 2.
[c]Between 1-2 years as reference group
[d]age*between 1-2 years as reference group
[e]Sensitization to at least one of 5 common aero- or 2 common food-allergens

© 2025 Hung CW et al. *JAMA Network Open*.

eTable 6. Early Life Characteristics by Atopic Dermatitis (AD) Remission Subgroups Among Children With Early Onset (ie, AD During the First 5 Years) in German MAS Birth Cohort

Characteristic	N	Persistent Phenotype		Remission Phenotype		Overall N = 658 n (%)	p-value[a]
		Substantial Adolescence Remission N = 116 n (%)	Partial Adulthood Remission N = 76 n (%)	Early School-Age Remission N = 291 n (%)	Early Childhood Remission N = 175 n (%)		
**Age of Onset**	658						<0.001
Age 1 year		77 (66%)	51 (67%)	125 (43%)	114 (65%)	367 (56%)	
Age 2 years		23 (20%)	13 (17%)	56 (19%)	61 (35%)	153 (23%)	
Age 3 years		11 (9%)	4 (5%)	43 (15%)	0 (0%)	58 (9%)	
Age 4 years		4 (3%)	7 (9%)	36 (12%)	0 (0%)	47 (7%)	
Age 5 years		1 (1%)	1 (1%)	31 (11%)	0 (0%)	33 (5%)	
**Parental Allergy Status**	627						0.14
One or two allergic parents		76 (68%)	55 (72%)	175 (63%)	96 (59%)	402 (64%)	
Non-allergic parents		35 (32%)	21 (28%)	101 (37%)	68 (41%)	225 (36%)	
Unknown		5	0	15	11	31	
**Early Allergic Sensitization** (during first 5 years of life)	321						0.013
Yes		55 (83%)	39 (87%)	94 (66%)	48 (71%)	236 (74%)	
No		11 (17%)	6 (13%)	48 (34%)	20 (29%)	85 (26%)	
Unknown		50	31	149	107	337	
**Sex**	658						<0.001
Female		64 (55%)	48 (63%)	132 (45%)	64 (37%)	308 (47%)	
Male		52 (45%)	28 (37%)	159 (55%)	111 (63%)	350 (53%)	

[a]Fisher's Exact test; Pearson's Chi-squared test

© 2025 Hung CW et al. *JAMA Network Open*.

eTable 7. Sensitivity Analysis of Association of Early Life Characteristics and Trajectories of Atopic Dermatitis (AD) Remission for the Children With Early Onset (ie, AD During the First 5 Years) in German MAS Birth Cohort

Characteristic	Early Childhood Remission[a] N = 175		Early School-Age Remission N = 291			Substantial Adolescence/Partial Adulthood Remission[b] N = 192		
	n (%)	RR	n (%)	Crude RR[c] (95% CI)	Adjusted RR[c] (95% CI)	n (%)	Crude RR[c] (95% CI)	Adjusted RR[c] (95% CI)
**Onset Between 1-2 Years** (vs onset between 3-5 years)	175 (100.0%)	Ref	181 (62.2%)	0.51 NaN	0.51 NaN	164 (85.4%)	0.48 NaN	0.59 NaN
**Onset Between 1-2 Years[b]** (vs onset between 3-5 years)	356 (76.4%) Ref[b]					164 (85.4%)	1.55 (1.09–2.21)	1.59 (1.12–2.26)
**One or Two Allergic Parents** (vs Non-allergic parents**)**	96 (58.5%)	Ref	175 (63.4%)	1.08 (0.93–1.26)	1.07 (0.92–1.25)	131 (70.1%)	1.28 (1.02–1.60)	1.23 (1.00–1.53)
**Female Sex**	64 (36.6%)	Ref	132 (45.4%)	1.14 (1.00–1.32)	1.14 (0.99–1.31)	112 (58.3%)	1.52 (1.24–1.86)	1.48 (1.21–1.81)
**Early Allergic Sensitization** (during the first 5 years of life)	48 (70.6%)	Ref	94 (66.2%)	0.94 (0.77–1.14)	0.94 (0.78–1.14)	94 (84.7%)	1.44 (1.00–2.08)	1.41 (1.00–1.99)
**Early Food Sensitization** (during the first 5 years of life)	32 (29.4%)	Ref	65 (32.7%)	1.06 (0.89–1.26)	1.07 (0.90–1.26)	65 (44.2%)	1.30 (1.06–1.60)	1.30 (1.09–1.56)
**Early Rhinitis** (during age 3-5 years)	33 (20.0%)	Ref	81 (30.2%)	1.21 (1.04–1.41)	1.19 (1.02–1.39)	74 (39.6%)	1.50 (1.25–1.81)	1.42 (1.19–1.69)
**Asthma at age 6 year**	1 (0.7%)	Ref	7 (3.0%)	1.42 (1.08–1.87)	1.45 (1.29–1.63)	17 (9.9%)	1.82 (1.55–2.12)	1.50 NaN
**Asthma at age 6 year[b]**	8 (2.1%) Ref[b]					17 (9.9%)	2.31 (1.71–3.12)	2.22 (1.70–2.89)

[a]Reference group for assessing AD remission subgroups and factors: parental allergy status, early allergic sensitization, early food sensitization, and early rhinitis

© 2025 Hung CW et al. *JAMA Network Open*.

[b]To avoid unstable estimates due to sparse data in certain subgroups, we combined “Substantial Adolescence Remission” and “Partial Adulthood Remission” into one group; for the similar reason, Early Childhood and School-Age Remissions were combined as a single reference group for factors: age of onset and asthma at age 6 year.
[c]Risk ratios were estimated using log-binomial regression. Parental allergy status was adjusted for sex; and sex was adjusted for parental allergy status; all other variables were adjusted for parental allergy status and sex.
Abbreviations: RR, risk ratio. aRR, adjusted risk ratio. CI, confidence interval

© 2025 Hung CW et al. *JAMA Network Open*.

eMethods 1.

Detailed categorization for age of AD onset according to various analyses

Analyses	Categorization
Description of study population	Dichotomous:<br>• Early-onset AD: when symptoms occurred for the first time between birth and five years<br>• Late-onset AD: when symptoms occurred for the first time after the age of five years
Risk of current AD using GLMM	Four groups:<br>• Onset between 1-2 years<br>• Onset between 3-5 years<br>• Onset between 6-15 years<br>• Onset between 20-30 years
Description of AD status during late adolescence and young adulthood (age 20 and 30 years)	Four groups:<br>• Onset between 1-2 years<br>• Onset between 3-5 years<br>• Onset between 6-10 years<br>• Onset between 11-15 years
AD remission prediction using GLMM (among children with early-onset AD)	Dichotomous:<br>• Onset between 1-2 years<br>• Onset between 3-5 years
Association of early life characteristics and AD remission trajectory (among children with early-onset AD)	Dichotomous:<br>• Onset between 1-2 years<br>• Onset between 3-5 years

© 2025 Hung CW et al. *JAMA Network Open*.

eMethods 2.

Detailed definition of early allergic sensitization and early-life allergy conditions

Participants were classified as early sensitized at a specific age if their specific IgE levels in serum were ≥0.35 IU/mL against at least one of five common aeroallergens (house dust mite, cat, dog, birch and grass pollen) or one of two common food allergens (cow's milk and hen's egg) at each respective age during the first five years of life with available serum samples. Subjects with only one missing time point, while not being sensitized at other time points, were defined as not early sensitized.

Allergic sensitization was considered a pathway covariate rather than a confounder; therefore, we evaluated its role by fitting two models (with and without adjusting for early allergic sensitization in the first 5 years) (eFigure 3, eTable 4). Food sensitization was further defined when participants' specific IgE levels in serum were ≥0.35 IU/mL against at least one of two common food allergens (cow's milk and hen'egg) at each respective age during the first five years of life with available serum samples. This variable was presented in sensitivity analysis only (eTable 7).

Rhinitis was defined as having a running, itchy or stuffed nose without a cold in the last 12 months. A composite dichotomous variable "early rhinitis" was created to indicate rhinitis occurrence between ages 3-5 years.

Asthma was defined as at least two of the following 3 criteria: doctor's diagnosed asthma ever, any indicative symptom in the last 12 months (i.e. wheezing, shortness of breath, dry cough at night), asthma medication in the last 12 months.

© 2025 Hung CW et al. *JAMA Network Open*.

eMethods 3.

Approaches for analyzing AD remission over time

Approach 1: A GLMM model of AD remission was developed to estimate the probability of AD remission across combinations of important early-life factors. The model included subject ID as random effect and dichotomized age of early-onset (1-2 vs 3-5 years), continuous age, sex, parental allergy status, early allergic sensitization (in the first 5 years), as well as the interaction between age of early-onset and age as fixed effect.

Approach 2: Growth mixture model (GMM) was applied to identify the AD remission trajectories. As an extension of GLMM, GMM is a parametric finite mixture modeling approach that assumes multiple mixed-effects models to identify unobserved subgroups (latent classes or phenotypes) within a population that share similar longitudinal trajectories. It estimates class-specific growth curves while allowing individual-level variation through random effects and can incorporate covariates to explain trajectory differences or class membership. (Nguena Nguefack, 2020) We modeled AD remission as a function of continuous time (age in years) as the only predictor without additional explanatory variables in the fixed part of the trajectory model. Individuals are assigned to a specific subgroup according to higher posterior probabilities. The number of classes was determined by a sufficient drop (elbow method) in Bayesian Information Criterion (BIC), a subgroup size over 10% of the total population, and the visual separation of the predicted trajectories using a plot. The group separation diagnostics are shown in the table and figures below.

	G	AIC	BIC	entropy	%class1	%class2	%class3	%class4	%class5	%class6
m1	1	8908.81	8917.79	1.00	100.00	NA	NA	NA	NA	NA
m2	2	7167.62	7190.06	0.82	69.45	30.55	NA	NA	NA	NA
m3	3	6831.89	6867.81	0.81	24.62	65.35	10.03	NA	NA	NA
m4	4	6538.33	6587.71	0.78	17.63	11.55	44.22	26.60	NA	NA
m5	5	6470.79	6533.64	0.79	7.90	26.60	41.19	17.33	6.99	NA
m6	6	6440.43	6516.74	0.74	8.97	4.26	37.08	13.68	9.42	26.60

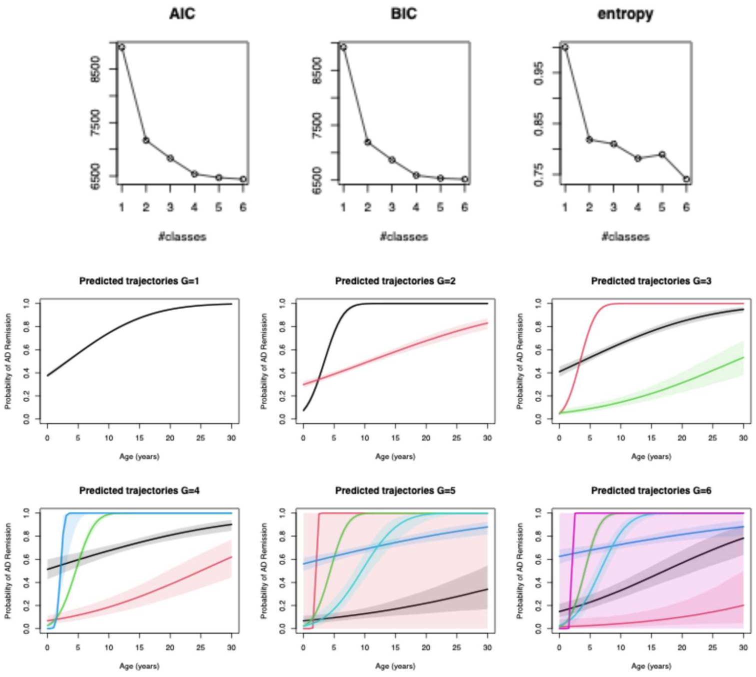

© 2025 Hung CW et al. *JAMA Network Open*.
