# Supplementary material for: Incidence and Remission of Atopic Dermatitis in a German Birth Cohort
Source: JAMA Netw Open. 2025 Dec 8;8(12):e2544324. doi: 10.1001/jamanetworkopen.2025.44324 (PMC12687101; doi:10.1001/jamanetworkopen.2025.44324)
Supplement: Supplement 2. — Data Sharing Statement [file jamanetwopen-e2544324-s002.pdf]

Data Sharing Statement

Hung. Incidence and Remission of Atopic Dermatitis in a German Birth Cohort. *JAMA Netw Open*. Published November 18, 2025. doi:10.1001/jamanetworkopen.2025.44324

Data
Data available: No

Additional Information
Explanation for why data not available: The data underlying this article cannot be shared publicly due to ethical/privacy restrictions. Access may be granted upon reasonable request to the corresponding author.
